# The Phytochemical Shikonin Stimulates Epithelial-Mesenchymal Transition (EMT) in Skin Wound Healing

**DOI:** 10.1155/2013/262796

**Published:** 2013-06-03

**Authors:** Shu-Yi Yin, An-Ping Peng, Li-Ting Huang, Ya-Ting Wang, Chun-Wen Lan, Ning-Sun Yang

**Affiliations:** Agricultural Biotechnology Research Center, Academia Sinica, 128 Academia Road, Section 2, Nankang, Taipei 115, Taiwan

## Abstract

Although various pharmacological activities of the shikonins have been documented, understanding the hierarchical regulation of these diverse bioactivities at the genome level is unsubstantiated. In this study, through cross examination between transcriptome and microRNA array analyses, we predicted that topical treatment of shikonin *in vivo* affects epithelial-mesenchymal transition (EMT) and the expression of related microRNAs, including 200a, 200b, 200c, 141, 205, and 429 microRNAs, in mouse skin tissues. *In situ* immunohistological analyses further demonstrated that specific EMT regulatory molecules are enhanced in shikonin-treated epidermal tissues. RT-PCR analyses subsequently confirmed that shikonin treatment downregulated expression of microRNA-205 and other members of the 200 family microRNAs. Further, expression of two RNA targets of the 200 family microRNAs in EMT regulation, Sip1 (Zeb2) and Tcf8 (Zeb1), was consistently upregulated by shikonin treatment. Enhancement of these EMT activities was also detected in shikonin-treated wounds, which repaired faster than controls. These results suggest that topical treatment with shikonin can confer a potent stimulatory effect on EMT and suppress the expression of the associated microRNAs in skin wound healing. Collectively, these cellular and molecular data provide further evidence in support of our previous findings on the specific pharmacological effects of shikonin in wound healing and immune modulation.

## 1. Introduction

Shikonin, a phytochemical derived from *Lithospermum erythrorhizon* (LE), has been shown to exhibit various biological and pharmacological activities [1, 2], including antioxidant [3], wound-healing [2, 4, 5], anti-inflammatory [4, 6, 7], and antitumor properties [8, 9]. Clinically, the use of shikonin dates back to the 5th century A.D. when it was used by Chinese herbalists for the treatment of burns, urticaria, and other allergic diseases [10]. Subsequently, it has been shown that the wound-healing activity of shikonin may result from its effect on the proliferation of fibroblasts, collagen fiber levels of the granuloma tissue [11], and proliferation of CD11b^+^ cells in such tissues [12]. Our group previously demonstrated that shikonin can confer a strong suppressive effect on gene expression of TNF-*α* at the promoter level in skin tissue [6] and a strong modulatory effect on specific mRNA splicing in human monocytes [7]. These findings suggest that the various pharmacological properties of shikonin may be due to a multifunctional and hierarchical effect on a spectrum of signaling genes at the genome and cellular levels.

In normal somatic tissues, epithelial-mesenchymal transition (EMT) is not only instrumental in wound healing but also in tissue fibrosis [13–15]. Downregulation of E-cadherin is one of the hallmarks of EMT and can lead to a concomitant increase in expression of specific mesenchymal cell markers (e.g., vimentin and fibroblast specific protein 1) as well as extracellular matrix-remodeling enzymes (i.e., matrix metalloproteinases) that are often observed together with a profound reorganization of the actin cytoskeleton [16, 17]. Previous studies have also shown that specific microRNAs in the microRNA 200 family are involved in the control of EMT activity in human epithelial cells, via repression of the translation of specific regulatory proteins, such as E-cadherin transcriptional repressors ZEB1 and ZEB2 (also known as Smad-interacting protein-1, SIP1) [18, 19]. Notably, we previously demonstrated that shikonin can effectively enhance the wound-healing process in severely damaged porcine skin tissues [20]. These observations and results together raised the hypothesis that topical treatment with shikonin may hierarchically modulate and facilitate the EMT process in skin tissues.

Profiling global gene expression patterns by DNA microarray analysis provides a useful approach for investigating complex biological phenomena [21], as we have previously shown for immune cell systems [22–24]. In addition, microRNAs, which are small ribonucleotides (~22 nt in length), act as key players in cellular differentiation and tissue development [25–28]. Characterization of the complex relationship between microRNAs and target mRNAs in shikonin-treated skin may thus assist in determining the molecular pathways and cell signaling processes involved in the process.

In this study we investigated the multifaceted effect of shikonin on mouse skin tissue *in vivo* at the transcriptome and the microRNA regulatory levels with an eye to future translational studies. We took advantage of the vast amounts of information obtained from decades of studies on shikonin-treated mammalian tissues [3, 4, 6–9, 11, 12, 20] and employed a network knowledge-based approach to analyze the genome-wide transcriptome activity *in vivo* for possible correlation with specific microRNA expression activities. We found that expressions of various EMT process-related microRNAs, such as microRNA-200a, -200b, -200c, -429, -141, and -205 were suppressed in skin after shikonin treatment. Consistent with these findings, histological results showed that shikonin induced EMT consistent cellular and tissue behavior in the epidermal layer during the process of wound healing. These findings provide cellular and molecular evidence supporting previous studies on the wound-healing [6, 20] and immune-modulatory activities of shikonins [6, 7] and have generated a new integrated strategy for investigating the mode of action of shikonins and other potent phytomedicines.

## 2. Materials and Methods

### 2.1. Mice

Female C57BL/6JNarl mice (National Laboratory Animal Breeding and Research Center, Taipei, Taiwan) were maintained under pathogen-free conditions on standard laboratory chow and water in the animal facilities of the Agricultural Biotechnology Research Center, Academia Sinica. All mice used in the experiments were 6–8 weeks old.

### 2.2. Treatment of Mouse Skin with Shikonin

Solutions containing 10 *μ*g/*μ*L shikonin (*Nacalai* Tesque, Kyoto, Japan) or 100 *μ*M phorbol 12-myristate 13-*acetate *(*TPA*; *Sigma*, St. Louis) in acetone were prepared immediately before use. Aliquots of 100 *μ*L of the test chemicals were pipetted onto the abdominal skin area of mice, immediately spread evenly, and allowed to air dry completely. Test skin treated with solvent (acetone) alone was used as a negative control. For each treatment, the test area was marked with an ink stamp encircling a surface area of 2.3 cm in diameter (i.e., 4.15 cm^2^). Animals were sacrificed at 4 or 24 hours after treatment, and treated or untreated skin samples were excised and frozen in liquid nitrogen for further RNA or protein expression analyses. For wound-healing experiments, 20 *μ*L shikonin solution or control solvent (acetone) was pipetted 3-4 mm beyond the margins of the wound (~40 mm^2^) on days 0, 2, and 4. The concentration of shikonin for topical application was 5 *μ*g/*μ*L (100 *μ*g/wound), which was selected based on our preliminary wound-healing experiment (data not shown).

### 2.3. Microarray and MicroRNA Array Analyses

For total RNA extraction, frozen skin samples were homogenized in liquid nitrogen. Subsequently, total RNA was extracted using TRIzol reagent (Invitrogen) according to the manufacturer's instructions and resuspended in 30 *μ*L of diethyl pyrocarbonate-treated (DEPC) water. A total of 8 hybridizations were performed for acetone-treated and shikonin-treated skin samples, and each test time point and treatment was analyzed in duplicate samples on separate chips. Standard Pearson correlation coefficients were used to determine the consistency of gene expression in each replicate array as previously described [24]. For microarray analysis, fluorescent aRNA (antisense RNA) targets were prepared from 2.5 *μ*g total RNA samples using OneArray Amino Allyl aRNA Amplification Kit (Phalanx Biotech Group, Taiwan) and Cy5 dye labeling (Amersham Pharmacia, Piscataway, NJ). Fluorescent targets were hybridized to the Mouse OneArray Whole Genome DNA microarray (Phalanx, Hsinchu, Taiwan) as previously described [29, 30], which contained 29,922 mouse genome probes, with Phalanx hybridization buffer using the Phalanx Hybridization System. After hybridization for 16 h at 50°C, nonspecific binding targets were washed away and the Cy5 fluorescent intensities for each spot were analyzed by GenePix 4.1 software (Molecular Devices, Sunnyvale). Data were analyzed using Spotfire software to determine whether a test gene was present or absent and whether the expression level of a gene in an experimental sample was significantly increased or decreased relatively to the control sample [24]. Changes in expression levels were evaluated as the averages of log_2_ [shikonin treatment/acetone treatment]. The microarray data have been deposited to the Gene Expression Omnibus database at NCBI (GEO; http://www.ncbi.nlm.nih.gov/projects/geo/) under the accession number GSE32694.

For microRNA array analysis, Febit's biochips (Geniom Biochip MPEA, mus musculus) were used. The probes were designed as the reverse complements of all mature microRNA sequences, which were published in the current Sanger miRBase release (version 14.0, September 2009) [31] for mus musculus. The probes on the chips were synthesized with intra-array replicates to increase the statistical confidence and to compensate for potential positional effects. RNA samples obtained from skin tissues treated with acetone or shikonin for 24 hours were subjected to a quality check to determine the quality and quantity of the sample RNA. The quality control was conducted using the Agilent 2100 Bioanalyzer and the RNA 6000 Nano Kit according to the manufacturer's instructions. For each array, test RNA was suspended in Febit's proprietary microRNA hybridization buffer (25 *μ*L per array). Hybridization was performed automatically for 16 hours at 42°C using the Geniom RT Analyzer. Following the labeling procedure, the biochips were stringently washed and the quantile normalization was performed according to the manufacturer's instructions [32, 33]. A total of 4 hybridizations were performed for acetone-treated and shikonin-treated skin samples, and each treatment was analyzed in duplicate on separate chips. In contrast to genomic DNA microarray analysis, the whole dataset microRNA (710 microRNAs) was systematically analyzed without filtering by “fold change” of expression level. Changes in expression levels were also revealed as averages of log_2_ [shikonin treatment/acetone treatment]. Combined analyses of the microarray and microRNA datasets were performed by using the MetaCore software to predict the modulatory effect of shikonin on skin tissue. The microarray data have been deposited to the database at NCBI (GEO; http://www.ncbi.nlm.nih.gov/projects/geo/) under the accession number GSE32695.

### 2.4. Immunofluorescence and Histological Staining

Skin specimens were fixed with 4% formalin and embedded in paraffin. For histological comparison, 6 *μ*m thick sections were then cut and stained with hematoxylin and eosin (H&E). For immunofluorescence staining, fixed tissue sections were initially immersed in boiling sodium citrate buffer (0.01 M sodium citrate buffer, pH 6.0) for 30 min (antigen retrieval step). Skin sections then were blocked with 5% nonfat milk and incubated with polyclonal anti-FSP-1 antibody (Millipore, 07-2274, 1 : 200 dilution), monoclonal anti-E-cadherin antibody (Epitomics, 4552-1, 1 : 100), or monoclonal anti-vimentin antibody (Santa Cruz, sc-21791-PE, 1 : 200) in 1% nonfat milk for 1 hour at room temperature. Sections were washed with PBS containing 0.1% Tween 20. To detect primary antibodies, some sections were incubated with fluorescein isothiocyanate-conjugated anti-mouse-IgG (Jackson, 111-095-003, 1 : 200) (for FSP-1). 4′,6-Diamidino-2-phenylindole dihydrochloride (1 *μ*g/mL) was used to stain nuclei. Fluorescence microscopy evaluation of immunostained tissue sections was performed using a Zeiss Axiovert 200 M microscope (Carl Zeiss, Heidelberg, Germany). Images were captured with a digital camera (Orca ER, Hamamatsu) and processed using Axiovision 4.6.3 (Carl Zeiss). The number of individual fluorescent spots as test cells was then scored for comparative analysis and the data were exported to Microsoft Excel.

### 2.5. Real-Time PCR Analysis

To verify the array results, 3 full-thickness skin samples per group were further analyzed as follows. For wound-healing experiments, skin tissues surrounding the wound area (1 cm in diameter) were prepared by removing the subcutaneous fat and connective tissue [34]. Real-time PCR reactions were performed using microRNA- and mRNA-specific primers for Zeb1; Zeb2; microRNA-200a, -200b, -200c, -141, -429, and -205. For mRNA analysis, complementary DNA (cDNA) was randomly primed from 2.0 *μ*g of total RNA using the Superscript reverse transcription kit (Invitrogen) according to the manufacturer's instructions. Reactions were incubated at 50°C for 50 min, and PCR amplification was carried out after denaturing at 95°C for 10 s. The following primers were used: mouse Zeb1, 5′-TGGCAAGACAACGTGAAAGA-3′ (forward), and 5′-AACTGGGAAAA TGCATCTGG-3′ (reverse); mouse Zeb2, 5′-TAGCCGGTCCAGAAGAAATG-3′ (forward), and 5′-GGCCATCTCTT TCCTCCAGT-3′ (reverse); mouse E-cadherin, 5′-AATGGCGGCAATGCAATCCCA AGA-3′ (forward), and 5′-TGCCACAGACCGATTGTGGAGATA-3′ (reverse); mouse Gapdh, 5′-AGGTCGGTGTGAACGGATTTG-3′ (forward), and 5′-TGTAGACCATGT AGTTGAGGTCA-3′ (reverse). Real-time PCR amplifications were performed using a LightCycler 2.0 Real-Time PCR System (Roche, Basel, Switzerland), and results were normalized to mouse glyceraldehyde 3-phosphate dehydrogenase (Gapdh) as an internal control. For microRNA analysis, 5 *μ*g of total RNA extracted from each test sample was reverse-transcribed into cDNA using the Mir-X miRNA First-Strand Synthesis Kit (ClonTech). The Mir-X miRNA qRT-PCR SYBR Kit (ClonTech) was then used to quantify microRNAs according to the manufacturer's instructions. The microRNA-specific forward primers contained the following sequences: mouse microRNA-200a, 5′-TAACACTGTCTG GTAACGATGT-3′; microRNA-200b, 5′-TAAT ACTGCCTGGTAATGATGA-3′; microRNA-200c, 5′-TAATACTGCCGGGTAATGATGGA-3′; microRNA-141, 5′-TAACACTGTCTGGTA AAGATGG-3′; microRNA-429, 5′-TAATAC TGTCTGGTAATGCCGA-3′; microRNA-205, 5′-TCCTTCATTCCACCGGAGTCTG-3′. A universal reverse primer was used for each microRNA according to the manufacturer's instructions. For microRNA quantification, the delta-delta *C*_*t*_ method was used to determine the level of each microRNA relatively to the level of U6 small nuclear (sn) RNA. Data are mean values ± S.D. of three independent experiments performed in triplicate.

### 2.6. Western Blot Analysis

For total protein extraction, acetone- (vehicle) and shikonin-skin samples were immerged in 500 *μ*L of lysis buffer (50 mM Tris-HCl (pH 7.4), 150 mM NaCl, 0.5% Triton X-100, 20 mM EGTA, 1 mM dithiothreitol, 1 mM Na_3_VO_4_, and appropriate protease inhibitor cocktail tablets) for 10 min. For isolation of total protein, mouse skin samples were excised, immediately placed in liquid nitrogen, and pulverized with metal beads (2 mm in diameter) at 4°C for 30 min. Lysates were centrifuged at 12,000 ×g for 20 min, and supernatant containing 30 *μ*g of protein was boiled in SDS sample loading buffer for 10 min before electrophoresis on a 8% PAGE BisTris gel (Invitrogen). After electrophoresis for 2 h, proteins in the gel were transferred to a polyvinylidene difluoride membrane (Novex, San Diego, CA), and the blots were blocked with 5% nonfat dry milk in PBST buffer (PBS containing 0.1% Tween 20) for 60 min at room temperature. The membranes were incubated overnight at 4°C with anti-MMP-9 antibody (1 : 50,000), anti-MMP-2 antibody (1 : 10,000), anti-E-cadherin antibody (1 : 3,000), or anti-vimentin antibody (1 : 3,000). Washed blots were incubated with a 1 : 10,000 dilution of HRP-conjugated anti-rat, anti-mouse, or anti-rabbit secondary Abs. Equal protein loading was assessed using mouse *β*-actin (Sigma). All antibodies were purchased from Abcam (Cambridge, UK). The protein bands were visualized on HyperMax film using the ECL system (Amersham Biosciences).

### 2.7. Wound Creation and Scoring of Wound Tissues

Cohorts of 8- to 12-week old C57BL/6 mice were used for the creation of physical wounds. All test animals were treated according to the guidelines outlined by the Institutional Animal Care and Use Committee. Two standardized full-thickness wounds, 4.5 mm in diameter, were made with a scalpel on mouse abdominal skins, one on each side of the midline. Eight mice were initially sacrificed at day 0, then a group of 16 mice (8 control and 8 shikonin-treated mice) were killed at each of the indicated time points (days 1–5). Therefore, in all, a total of 88 mice were randomly assigned to either the control group or the shikonin-treatment group. Digital images were taken on the day of surgery and each subsequent day. For scoring of wound healing, the wound areas (in mm^2^) were calculated from wound perimeter tracings and were compared using Image Pro Plus 6.0 software (Media Cybernetics, Maryland). Wound areas were normalized by setting the day 0 wounding level as 100%; relative wound areas on the subsequent days were expressed as a percentage of the day 0 value [35]. After scoring of the wound area, three mice from each group (*n* = 8) were subjected to immunofluorescence observation and the other five mice underwent RNA expression study.

## 3. Results

### 3.1. Histological and Physiological Changes in Mouse Skin Tissues in Response to Topical Treatment with Shikonin

**Figure 1:**
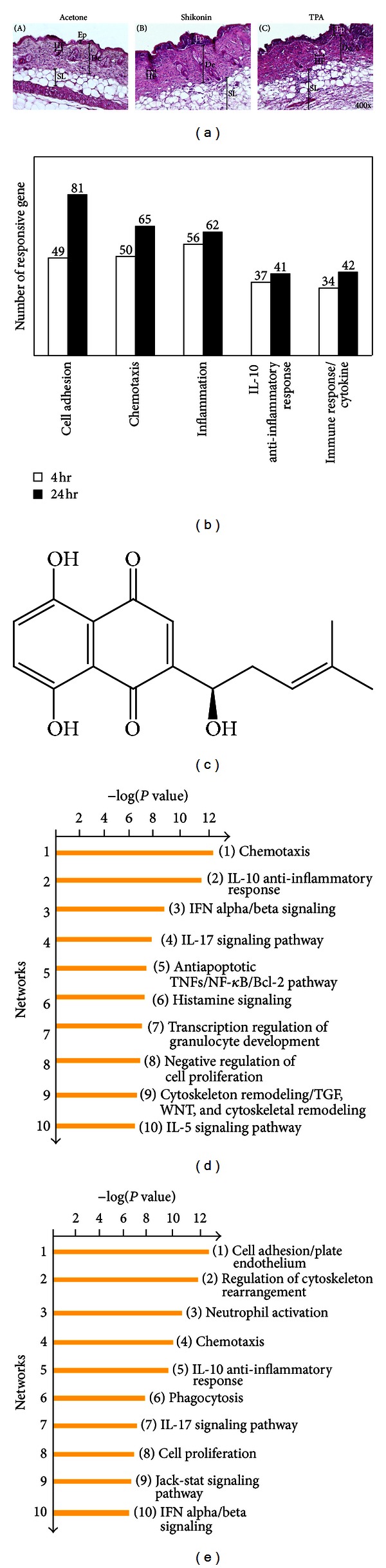
Histological analysis shikonin-treated mouse skin and grouping of genes that are responsive to shikonin treatment. (a) The histological characteristics of acetone- (vehicle control), shikonin-, and TPA-treated skin were revealed by H&E staining. Test mice (3 animals per group) were topically treated with (1) 100 *μ*L acetone, (2) shikonin (3.5 mM in 100 *μ*L acetone), or (3) TPA (10 nM in 100 *μ*L acetone) for 24 h. Black bars mark the thickness of the epidermal layer (Ep), dermis (De), subcutaneous layer (SL; also known as hypodermis), and hair follicles (HF). The dotted line in panel 3 indicates a terminally damaged boundary line between the epidermal (stratum spinosum) and dermal layers. (b) Shikonin-responsive genes in skin, as compared to acetone- (vehicle control) treated skin samples, were grouped according to their known molecular, cellular, or biochemical functions. Classified gene groups were then listed according to the abundance in number of responsive genes in skin tissue treated with shikonin for 4 or 24 h. (c) Structure of the naphthoquinone, shikonin. (d and e) GeneGo Pathway Maps processed by MetaCore software analysis. By comparing the −log⁡ [*P* value] of different gene-clustering groups, the top ten responsive processes with specific cellular or molecular functions were predicted for evaluating the effect of shikonin. The orange bars indicate the calculated values generated from 4 h (d) and 24 h (e) datasets, respectively.

We initiated this study by evaluating the histological and physiological effects of shikonin on mouse abdominal skin.  Figure 1(a) shows the histological features of typical skin biopsy samples of four representative test mice. A total of 5.3 cm^2^ of skin area was treated for 4 or 24 hours with 100 *μ*L acetone containing 10 *μ*g/*μ*L (3.5 mM) shikonin or 100 *μ*M 12-O-tetradecanoylphorbol-13-acetate (TPA), a well-known inflammatory agent. TPA stimulation was used as a positive control for an inflammatory condition in skin tissue. We observed a much thicker epidermal tissue with up to a 5-fold increase in thickness (shown as black bars) in shikonin-treated skin tissues than control skin tissues. The epithelium of skin tissues in the acetone- (vehicle control) treated group exhibited only 2-3 cell layers. Underneath the epithelium, a high population of cells infiltrated into the dermis layer in shikonin-treated skin tissues (Figure 1(a)—(B)) as compared to those treated with acetone (Figure 1(a)—(A)). These results suggest that shikonin can enhance the tissue permeability or cell chemotaxis in test mouse skin tissues, indicating that shikonin can initiate its cellular or physiological effect at a very early stage during the wound-healing process, that is, at 4 h after treatment)”. In addition, disorganization and an increased number of fat cell layers were readily observed in shikonin-treated skins (Figure 1(a)-(B)). In mouse skins topically treated with TPA, epidermal cell layers became swollen and thickened, and various cell types penetrated into the stromal/dermal/fat tissue layers (Figure 1(a)-(C)), in a similar fashion as observed for shikonin-treated skin tissue (Figure 1(a)-(B)). However, the shikonin-treated skins still maintained a distinctive border (stratum basale) between the epidermal layer and the dermal layer; by contrast, in TPA-induced inflamed skin, no boundary between the epidermal and dermal tissues could be detected. These specific and different physiological effects on multiple cell layers of normal skin tissue suggest that although the shikonin-treated skins exhibited some inflammation-associated cellular characteristics, the treatment apparently did not cause damage at the tissue level. These results support previously reported pharmacological activities of the shikonins and the traditional medical use of shikonin-containing *Lithospermum erythrorhizon* plants in skin wound-healing [4–6].

### 3.2. Shikonin Treatment Results in Differential Gene Expression in Mouse Skin Tissues

In order to systematically evaluate the effects of shikonin on mouse skin tissues, we next compared the gene expression profiles in shikonin-treated versus acetone-treated skin tissues at different time points. Total RNA samples were collected at indicated time points for transcriptome and microRNA array analyses as described previously [22–24]. Only genes showing at least a 2-fold change in the level of RNA transcripts in two independent experiments were considered as significant for further analysis. Differences in gene expression levels were calculated by dividing the signal intensity values obtained from shikonin-treated skin tissues by those from acetone-treated tissues. Gene expression profiling analysis showed that a total of 2,300 and 3,930 genes had markedly changed expression levels after 4 hours and 24 hours of pretreatment with shikonin, respectively. By comparing and grouping the number of shikonin-responsive genes using the MetaCore program, we found that shikonin treatment can result in 5 major cellular/physiological effects with known molecular or biochemical functions (Figure 1(b)). This gene-clustering analysis showed that the top five gene functional groups significantly modulated through shikonin stimulation, either at 4 or 24 h after treatment were cell adhesion (cytoskeleton and cell movement), chemotaxis, inflammation, IL-10-mediated anti-inflammation, and cytokine-mediated immune response. These responses suggest that shikonin treatment induces an integrated tissue-wide response involving cell chemotaxis, attachment, and cellular immune responses.

Next, these responsive genes were grouped and classified according to the *P* value of the hypergeometric intersection, which indicates the “trend” or the “consistence” of a test dataset in gene-clustering analysis. A lower *P* value of the entity means higher relevance to the dataset, which appears in the higher rating of the entity [36]. By comparing the −log⁡ [*P* value] level in the different gene clusters, different gene groups can be assigned according to their known cellular and molecular processes, using the MetaCore software. This analysis predicted a list of top ten cellular and molecular processes that may be regulated or affected in skin by topical treatment with shikonin, at 4 (Figure 1(d)) or 24 hours (Figure 1(e)) after treatment. It is important and interesting to note here that the regulation of immunological signaling networks and the control of cell or tissue development signals represent over 80% of the shikonin-responsive gene expression activities. Among them, two are specifically related to cell adhesion or cytoskeleton remodeling. In addition, several key cytokines, including IL-10 and IL-17, were also predicted to play a role in defined cytokine-mediated immune activities in response to shikonin (Figures 1(d) and 1(e)). Interestingly, our analysis further revealed a relationship between shikonin stimulation and activities of specific cell types, such as transcription regulation of granulocyte development and neutrophil activation. These results suggest that shikonin treatment of skin may induce specific growth or differentiation activities according to cell type and may subsequently facilitate the tissue remodeling at the organ level that we had previously observed in mouse and pig skins [6, 20].

### 3.3. Combination of Transcriptomic and MicroRNA Array Analyses for Evaluating Signaling Networks Modulated by Shikonin in Skin Tissue

To examine possible hierarchical regulation of the effects of shikonin on gene expression networks at the transcriptome level and to determine the main factors that may coordinate high-level regulation of the stimulatory effects of shikonin on skin tissues, we extended our investigation by combining the use of microRNA array and the transcriptome microarray. In this test, the microRNA expression values for a total 710 microRNAs were obtained from output data. Initial experimental preparations yielded Pearson's correlation values of 0.97 and 0.92 for test biological sample replicates of shikonin- and acetone-treated skins, respectively, confirming the validity of the assay stringency. All microRNAs identified from the normalized data of this analysis were then employed to identify candidate target genes in subsequent studies.

**Figure 2:**
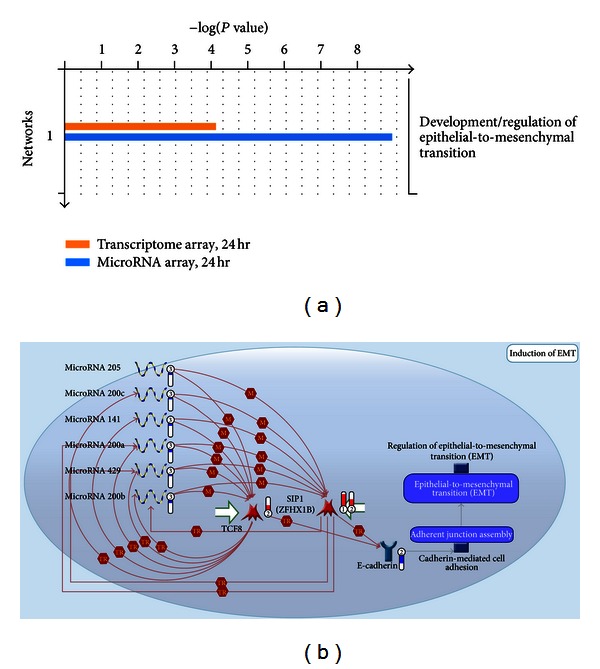
Combinational analysis of transcriptome/DNA microarray and microRNA array datasets to predict possible effects of shikonin on mouse skin tissue. (a) Functional analysis and prediction by “GeneGo Pathway Maps” processed by MetaCore software. By analyzing the −log⁡ [*P* value] value of different gene-clustering groups in the 24 h treatment datasets obtained from the transcriptomic array and microRNA array analyses, the regulation of the EMT process was predicted to be the major physiological response to shikonin stimulation. The orange and blue bars indicate the calculated value output from transcriptomic array and microRNA array analysis, respectively. (b) Putative signaling networks (microRNA-dependent inhibition of EMT) involved in the modulatory effect of shikonin on skin tissue were predicted from the MetaCore software. In this map, a prototypical cell was constructed from six representative microRNAs and three differentially expressed genes that respond coordinately to an *in vivo* treatment with shikonin for 24 h. Experimental data from transcriptomic array and microRNA array analyses are linked to and visualized on the maps as thermometer-like symbols. Red and blue scales indicate the upregulated and downregulated gene expressions (microRNAs or mRNAs). M: microRNA binding. TR: transcription regulation. TCF8 (Zeb1): transcription factor 8. SIP1 (Zeb2; Zfhx1B): Smad-interacting protein 1.

Having analyzed the *in vivo* microRNA expression profiles of cells in skin tissues, we observed that expression levels of a number of microRNA species were consistently regulated after treatment with shikonin for 24 hours. These include the expression of six microRNAs of the microRNA 200 family, microRNA-200a, -200b, -200c, -141, -429, and -205, which were all downregulated (see the left blue bar-containing thermometers in Figure 2(b)). Consistent with this result, the transcriptomic array analysis also showed that the gene expression levels for Zeb1 (*Tcf8*) and Zeb2 (*Sip1*; *Zfhx1b*) were upregulated after shikonin treatment for 24 hours (see the red bar-containing thermometers in Figure 2(b)).  Figure 2(a), obtained from the *GeneGo Process* of the MetaCore software, shows the hypothetical or candidate networks of EMT processing revealed by our combined analyses of the representative genes and the microRNAs in coordination as a response to shikonin stimulation. This analysis predicted that the EMT process is the only pathway strongly modulated by shikonin treatment of test mouse skin tissue; however, this conclusion was only obtained when we performed the combined analyses of the datasets obtained from both the transcriptome and the microRNA expression profiles (Figure 2(a)). Taken together, our results therefore strongly suggest that shikonin treatment has a potent suppressive effect on the expression of different members of the microRNA 200 family, and the resultant cascade at the RNA transcript level efficiently activates the EMT process through coordinated and potent activation of the Zeb1 (*Tcf8*) and Zeb2 (*Sip1*) gene expressions, the master regulators of the EMT activity.

### 3.4. Induction of EMT Features in Shikonin-Treated Skin Tissue

**Figure 3:**
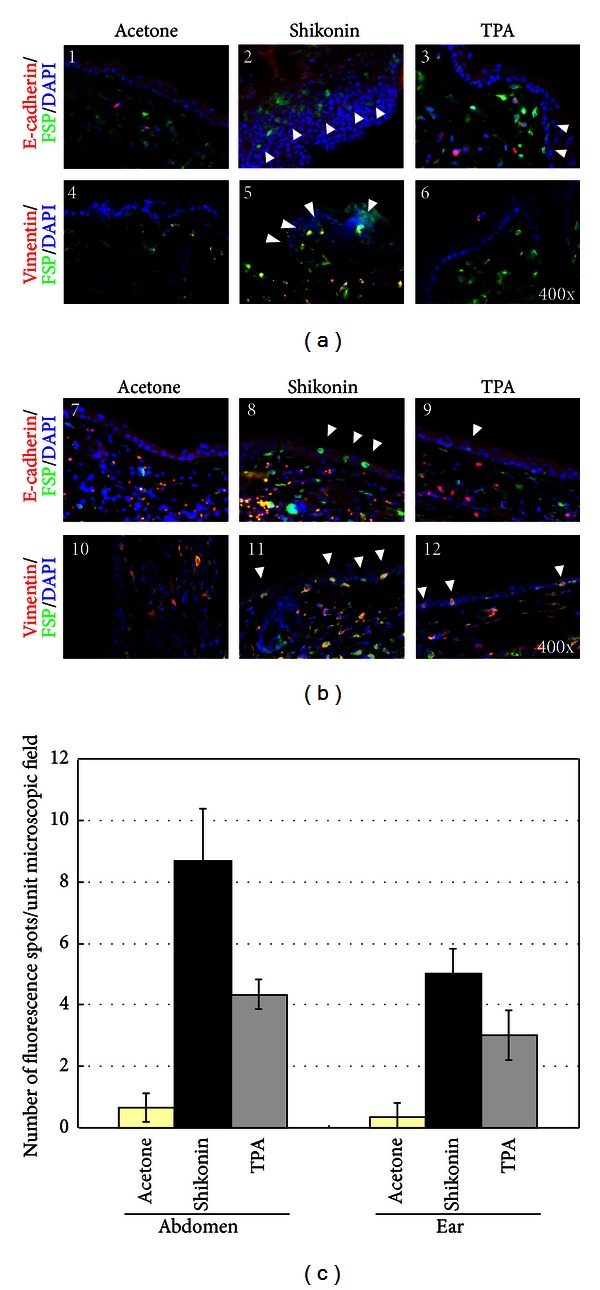
Gain of mesenchymal features by epithelial cells in mouse abdominal and ear skin tissues treated with shikonin. (a) At 24 h after treatment, (1) acetone-, (2) shikonin-, and (3) TPA-treated abdominal skin tissues were stained for the presence of E-cadherin (red), FSP1 (green and indicated with arrowheads), and DAPI (blue) to reveal the appearance of mesenchymal cells in epidermis. The presence of vimentin (red) and FSP1 (green) double-stained cells (yellow and indicated with arrowheads) further indicates that these epithelial cells underwent the EMT process in test skin tissues (4–6). (b) Analogous analysis was performed on treated ear epidermis (7–12). (c) Statistical comparison of EMT activity was performed by scoring the number of yellow fluorescence spots (vimentin^+^/FSP1^+^) in (a) and (b). Values shown are the mean ± SEM of three experiments. The significance of differences was analyzed by one way ANOVA (*P < 0.05, **P < 0.01, and ***P < 0.001).

As a followup, the effect of shikonin on the specific cellular features of the EMT process was evaluated in mouse skin using immunofluorescence staining assay. It is known that epithelial cells undergoing the EMT process, that is, those cells that are localized above the basement membrane zone (BMZ) that have gained specific mesenchymal cell markers, are fibroblast-specific protein 1 (FSP1) and/or vimentin positive [13, 16, 17]. In our study, this layer of epithelial cells was characterized by E-cadherin expression (red staining in Figures 3(a)—1; to 3 and 3(b)—7 to 9). The EMT features in TPA-treated skin tissues were detected as a positive control for inflamed skin tissue (Figure 3(a)—3 and 6; Figure 3(b)—9 and 12). A comparison of shikonin- and acetone-treated skin tissues showed that shikonin-treated skins contained a much higher population of epithelial cells positively stained for FSP1 and/or vimentin (Figure 3(a)—2 and 5; Figure 3(b)—8 and 11). By contrast, the surrounding epithelial cells clearly lacked these mesenchymal markers. In agreement with the result from H&E staining of abdominal skin tissue, the thickness of epithelial cell layers was drastically increased in skins treated with shikonin (Figure 1(a)). Statistical analyses of EMT activity clearly showed that shikonin treatment could activate the EMT process in abdominal skin tissues (Figure 3(c)). These results demonstrate the specific stimulatory effect of shikonin on the EMT process in test mouse skins, strongly supporting the results obtained from the microRNA array and transcriptome analyses.

**Figure 4:**
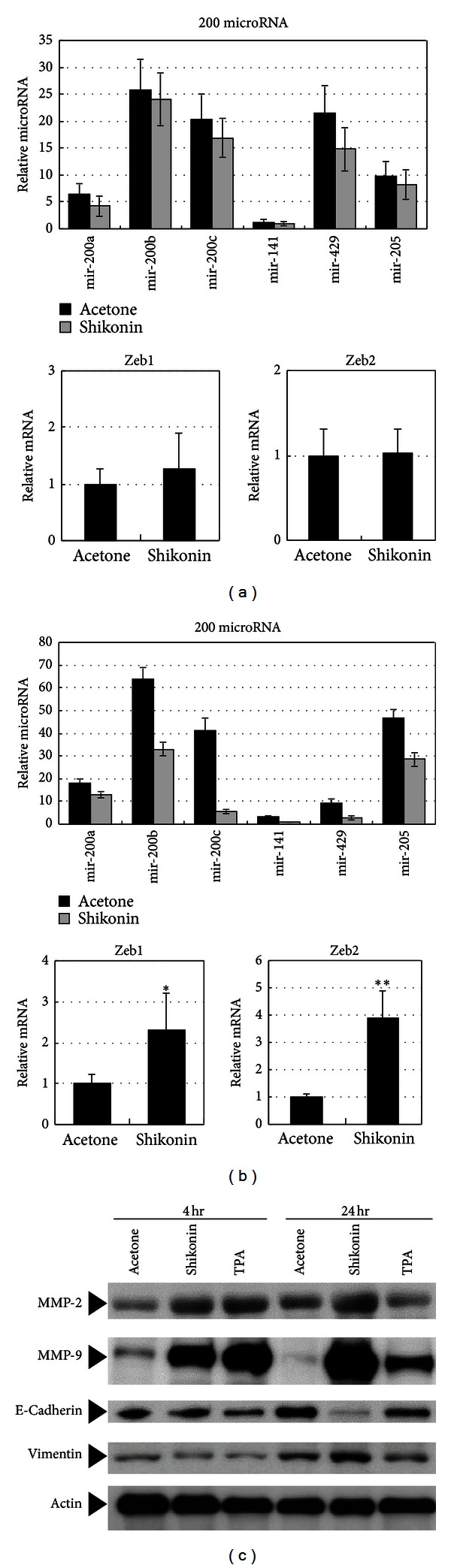
Validation of the EMT activity-related expression of microRNAs and proteins. The expression levels of some key mesenchymal-associated microRNAs, that is, mir-200a (microRNA-200a), mir-200b (microRNA-200b), mir-200c (microRNA-200c), mir-141 (microRNA-141), mir-429 (microRNA-429), and mir-205 (microRNA-205), as well as Zeb1 and Zeb2 mRNAs in epidermal cells treated with shikonin for 4 h (a) and 24 h (b) were determined by real-time PCR analyses. Values shown are the mean ± SEM of three experiments. The significance of differences was analyzed by one way ANOVA (*P < 0.05; **P < 0.01). (c) Expression of E-cadherin and other mesenchymal-associated markers, MMP2, MMP-9, and vimentin, were measured by Western blot analysis. *β*-actin was used as gel loading control. Data are representative of triplicate experiments.

The effect of shikonin on EMT features was also evaluated in ear skin tissues (Figure 3(b)). We found that some cells exhibiting mesenchymal cell markers were also detectable in the ear skin tissues treated with shikonin. However, the level of change in EMT activities was not as obvious as that detected in abdominal skin (Figure 3(a)—2 and 4). The reason for the difference may be the thickness of the epithelial layers in these two different skin tissues. Shikonin treatment did not result in a significant increase in skin thickness in ear skin tissue, contrary to the results seen in abdominal skin. It is important to note here that ear skins have traditionally been used as a popular model for evaluating different pharmaceutical effects of candidate drugs or therapeutics. Our data suggest that the potency or specificity of shikonin-induced EMT activity may depend on skin tissue and the constituent cell type. 

### 3.5. EMT-Related MicroRNA and Protein Expressions in Skin Tissue Are Regulated by Stimulation with Shikonin

Previous studies have shown that expression of microRNA-200 family members and microRNA-205 in particular is necessary for the maintenance of the epithelial phenotype [18, 19], that is, the reverse of EMT activity; thus, we further validated the RNA transcript and microRNA array expression results. The expression levels of EMT process-related mRNAs (Zeb1 and Zeb2) and microRNAs (microRNA-200a, -200b, -200c, -141, -429, and -205) were quantitatively determined by real-time PCR (RT-PCR). In comparison with the vehicle-control skin samples, the observed decrease in the expression levels of specific microRNAs in shikonin-treated skin, although seeming following a similar pattern, was insignificant at 4 hours after treatment (Figure 4(a)). However, at 24 hours after treatment, the expression of these microRNAs was significantly suppressed by shikonin treatment (Figure 4(b)). Consistent with this data, the levels of Zeb1 and Zeb2 mRNAs were also significantly increased in skin tissues treated for 24 hours with shikonin. Taken together, our data suggest that suppression of the expression of these microRNAs plays an important role in the shikonin-induced EMT process *in vivo* in mouse skin. Interestingly, the suppression of the expression of some microRNAs (i.e., microRNA-200a, -200b, and -205) was not statistically significant as conventionally measured by “fold change.” However, when the microRNAs are viewed as a whole, a clear trend in expression could be seen. Based on the pattern of coordinated regulation, our data does support that such less than “substantial fold change” for individual microRNAs, but good alliance in the expression trend as coordinated with across-the-board alteration may confer a good regulatory network for gene expression controls. Our study may thus also indicate the need for such systematic analyses of trends in microRNA expression profiles.

As shown in Figure 4(c), the expression levels of some mesenchymal-associated protein markers such as MMP2, MMP-9, and vimentin were substantially increased in cells of skin treated with shikonin for 24 hours as compared to those in cells obtained from control skin samples, thus validating the shikonin-induced EMT activity in abdominal skin cells. In addition, we observed significant downregulation of E-cadherin expression, another hallmark of EMT, in shikonin-treated skin tissue, especially at 24 hours after treatment. These data are consistent with the mesenchymal features that we observed histologically in tissue sections. 

### 3.6. Epithelial Cells Located in the Wound Margin of Shikonin-Treated Skin Tissues Acquire Mesenchymal Cell Phenotypes

**Figure 5:**
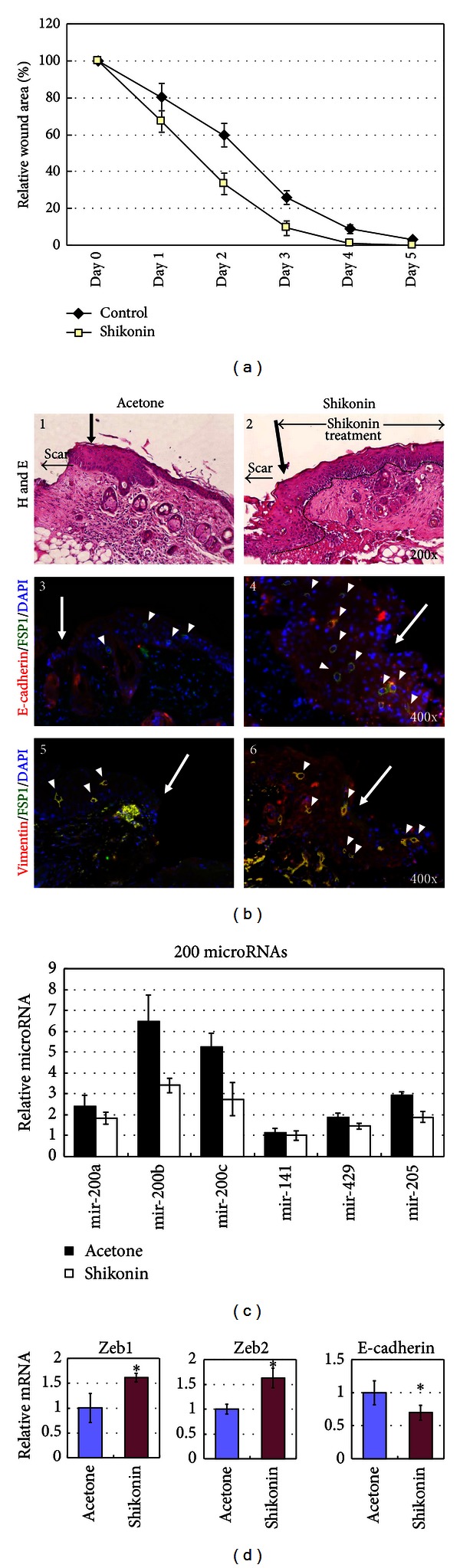
Characteristics of EMT in shikonin-treated wound healing in mouse skin tissues. (a) Wound closure rates of skins subjected to different treatments (shikonin versus control). The data represent the mean ± SEM of six mice. At 2 d after treatment, histological and immunohistochemical characteristics of acetone- (1) and shikonin-treated skins (2) were analyzed. Long arrows indicate the original edges of each wound. The dotted line in panel 2 indicates the terminally damaged boundary line between the epidermal and dermal layers. (b) Tissue biopsy samples from each test group were also stained for the expression of E-cadherin (red), FSP1 (green), and DAPI (blue). Triangle arrows indicate the presence of mesenchymal cells in the epidermis (3 and 4). The presence of vimentin (red) and FSP1 (green) double-stained cells (yellow and indicated with arrowheads) further indicates that these epithelial cells have undergone the EMT process in test skin tissues (5 and 6). (c) Changes in expression levels of mesenchymal-associated microRNAs, including mir-200a, mir-200b, mir-200c, mir-141, mir-429, and mir-205, as well as the Zeb1, Zeb2, and *E-cadherin* mRNAs (d) in shikonin-treated skin wound, as measured by real-time PCR analyses. The significance of differences was analyzed by one way ANOVA (*P < 0.05 and **P < 0.01).

To evaluate the enhancing effect of shikonin on tissue repair in skin wounding, aliquots of 20 *μ*L shikonin solution (5 *μ*g/*μ*L) or control solvent were topically applied to the area surrounding the test wound. Wound closure was measured as the area of epidermal closure (mm^2^) from the initial wound after treatment, and the rates of wound closure (area of granulation tissue remaining exposed) were measured for different treatments (Figure 5(a)). The addition of shikonin (100 *μ*g/wound) significantly accelerated wound-healing activity, especially during the early repair phase (0–2 d), as compared with the acetone-treated mouse skins. This result suggests that topical application of shikonin can increase the rate of wound closure kinetics for skin wound healing.

During the reepithelization phase, epithelial cells in the wound margin tissues can be mobilized through gain-of-mesenchymal features during the wound repair process [34]. Our results above led us to suggest that partial EMT activity is induced by topical application of shikonin onto wounded skin tissues. Therefore, we evaluated mouse skin tissues around the acute wounds during the phase of re-epithelialization. Skin biopsy samples were harvested between 0 and 5 days after the creation of aseptic wounds. As shown in Figure 5(b) through H&E staining, the active migratory tongue of epidermal cells penetrated into the wound tissue bed under the scab at the wound margin (black arrows) at 2 days after stimulation. In these specimens, we observed that many epithelial cells undergoing the EMT process indeed gained mesenchymal markers, such as FSP1 and/or vimentin (Figure 5(b)—3 to 6). A comparison of the shikonin- with acetone-treated skin wounds showed that the migration tongues of shikonin-treated skins contained a significantly higher population of the epithelial cells that were stained positively for FSP1 or vimentin (Figure 5(b)—4 and 5). In addition, expression levels of the 200 family microRNAs in shikonin-treated skin surrounding the wound were also significantly lower than those in the untreated skin, at 2 days after treatment (Figure 5(c)). Consistently, the levels of Zeb1 and Zeb2 mRNAs were also significantly increased in the shikonin-treated skin wounds (Figure 5(d)). Taken together, the results shown in Figure 5 are consistent with the data obtained from normal skins which were not subjected to wound creation (Figures 3 and 4). In summary, our data acquired by integrating multiple experimental approaches suggest that shikonin can confer a highly efficacious stimulatory effect on the EMT process, via which epithelial cells can effectively acquire the mesenchymal machinery features to guide cell migration and tissue repair.

## 4. Discussion

Using a combination of mRNA and microRNA expression profile analyses, we investigated the *in vivo* effects of the medicinal phytochemical shikonin, on cellular, histological, and immune-modulatory activities in mouse skin tissue. In the present study, although the changes in expression levels of some 200 family microRNAs were modest (e.g., 1.4- to 1.8-fold change) in response to shikonin treatment for 24 hours, the high degree of consistency in the trends in expression strongly suggests that these microRNA species together may play important roles in suppressing specific target genes and result in hierarchical control and onset of a shikonin-induced EMT process and the subsequent cascade activities. These results further suggest that because the role of microRNAs in regulation of gene expression is often sequence dependent and virtually always involves coregulation by several microRNA or siRNA components [37–40], measurement of “fold change” of their expression levels may not be a wholly satisfactory method to evaluate and filter responsive microRNAs. Based on the current findings, we hence further suggest that our strategy in combining the profiling and clustering of microRNA expression activities with specific transcriptome activities is a systematic and logical approach for evaluating the effect of certain phytocompounds on specific cellular or physiological functions that are regulated in a coordinated and hierarchical manner. This combinational approach may be superior to transcriptome or microRNA profiling alone for the evaluation of phytomedicines.

 Previously, the skin tissue obtained from the mouse ear has been employed as a mouse skin model applied for treating various dermal diseases or testing drug delivery efficacy. The advantages of this tissue origin may include its relatively simple composition of epidermal or dermal cell layers and the ease with which it can be obtained from test mice. However, the interactive relationships between skin and other tissue layers in the ear can be drastically different from the relationships in other body parts, which often exhibit a more complex interaction with the underneath substratum tissues. In this study, we found that topical application of shikonin could markedly increase the thickness of both the epidermal and the dermal layers of the abdominal skin (Figure 1(a)) but not ear skin. In addition, the level of shikonin-induced EMT activity in ear skin (Figure 3(b)) was much less obvious than that in skin tissues of the abdomen (Figure 3(a)) or thigh (data not shown). These results suggest that the lower EMT activity we observed in ear skin may be due to its relatively simple tissue composition. Such tissue diversity in different skins of the body should be explored for future development of skin remedies or healthcare agents. 

 The anti-inflammatory effects of shikonin have been investigated in a spectrum of physiological, molecular, and cellular studies [4, 6, 7, 41, 42]. In this study, our data also show that some IL-10-mediated anti-inflammation response-related genes were activated after shikonin treatment for 24 hours (Figure 1(b)). However, a comparison with TPA-induced inflamed skin in this investigation revealed that some proinflammatory histological properties and molecular mediators are also induced in shikonin-treated abdominal skin tissues. These features include the changes in skin thickness (Figure 1(a)), expression of some inflammation-related genes (Figure 1(b)), and expression of some inflammation-related protein molecules, such as MMP2 and MMP9 (Figure 4(c)). The differences between some of our results and previously reported results may reflect the different skin models we used. On the other hand, it has become increasingly evident that different proinflammatory stimuli may differentially affect gene expression profiles and/or genome activities. Similarly, different anti-inflammatory agents may also act differently against different inflammatory conditions. In both cases, interactive and interactive signalings apparently can generate specific tissue and cellular environments for dealing with and responding to different physiological processes or stresses, such as inflammatory activities, stress response, wound repair, and tissue remodeling [43–45]. We previously showed, using time-course experiments, that shikonin can greatly affect human monocyte/dendritic cells involving groups of anti-inflammatory and proinflammatory cytokines/chemokines in “cross-talk” and “overlapping” manner [46]. These findings together with the current results suggest that shikonin might be a promising molecular agent for treatment of various diseases or epithelial tissue damages. However, the optimal dosage for different skin tissues (e.g., ear versus abdomen) for potential future clinical use of shikonin remains to be identified.

The highly effective wound-healing activity of shikonin has been demonstrated in a number of *in vivo* studies using different animal models [6, 12, 20]; however, the molecular or cellular mechanisms and the derived guidance for supporting this therapeutic use of shikonin are not known in detail. In fact, some of the reported effects of shikonin, including anti-inflammatory [4, 6, 7] and antiangiogenesis activities [4, 42], may be considered physiologically or biochemically contradictory to its wound-healing activity. In the present study, the specific effect of shikonin on the EMT process and on related microRNA expression has provided us with valuable information and new insights into the wound-healing activities of shikonin. In wound-healing research, EMT has been shown as a very important cell transdifferentiation process for both acute and fibrotic cutaneous wound-healing activities in human skin [34]. On the other hand, because EMT activity also plays a role in carcinogenesis and neoplasia, there may be concern that shikonin-induced EMT activity could lead to carcinogenesis of local tumors. In 2009, EMTs were classified into three different biological subtypes based on various biological contexts [47, 48]. Among these subtypes, the EMTs associated with wound healing, tissue regeneration, and organ fibrosis are classified as type 2, whereas EMTs assigned for activities in development of localized tumors are classified as type 3. Although the specific signals that delineate the EMTs in the three discrete settings are not totally clear, it is known that the features of EMTs (expression of FSP-1, vimentin, and cell proliferation activity) are transient and reversible for the type 1 and type 2 EMT processes, but not for type 3 EMT. In this study, we observed that specific EMT features were readily detectable during the wound-healing process of mouse skin tissues (data not shown). Moreover, to our knowledge studies indicating the “carcinogenesis-promoting” activity of shikonin have not been previously reported. Taken together, we therefore consider that the beneficial effect of shikonin on skin wound-healing is mainly related to the type 1 EMT process. To add to our current findings, in the future we will investigate the specific targets of shikonin and the mechanistic roles of the specific molecular or cellular components at the tissue/organ level in shikonin-enhanced wound-healing activity.

## 5. Conclusions

In conclusion, we characterized the specific effects of shikonin in skin wound-healing activities at the molecular and cellular levels by probing the cell transdifferentiation processes and microRNA regulation. We consider that comprehensive analyses using different omics approaches in our *in vivo* experimental system can be usefully employed for integrated evaluation of the pharmacogenomic activities of the shikonins and other phytochemicals from various traditional medicinal herbs that are routinely used as remedies.
